# Dangerous Classes

**Published:** 1863-01

**Authors:** 


### Art. XI.—DANGEROUS CLASSES.

"It is the fear of want which rendereth any animal greedy or ravenous. And beside this fear, there is a pride in man which maketh him esteem it a glory to excel his fellow-creatures in pomp and excess. The laws of Utopia leave no room for these feelings."—Sir Thomas More, *Utopia,* p. 71.

Frank-pledge was a sort of legal acknowledgment that there was a dangerous or a doubtful man in every ten. The members of a tything were perpetual bail for each other. Michelet gives you the idea that for ages in France the populace naturally divided into two classes—an armed police and the defenceless suspected. Hallam says the poor very early felt the necessity of selling themselves to some bellicose baron for subsistence and protection. Fletcher of Saltoun recommends that the multitudes of mendicants, desperadoes, and idlers in his own time, the débris of war and commotion, and who threatened or interfered with the peaceable and industrious members of the community, should be deported as slaves. This was done when the children's Crusade broke up. About twenty years ago a benevolent Belgian dedicated a huge book, of seven hundred pages, to the specific subject of the Dangerous Classes in great cities.* His essays are devoted to ragpickers, gamblers, prostitutes, vagabonds, impostors, pickpockets, thieves, liberated convicts; and, nauseated and discouraged by his own revelations, he says towards the close of his work: " Obligé par la nature de mon sujet de fouiller dans les replis les plus cachés et les plus fangeux de la société, et de mettre en relief les vices et les méfaits de tout genre qu'on y projette ou qui y fermentent, je n'ai pu m'empêcher de donner aux tableaux que j'ai esquissés, aux considérations que j'ai exposées, une forme ou une couleur à la fois triste et severe" (p. 610.) More recently it has been calculated that the pariah constituents of the population in London might amount to 120,000 gamblers, thieves, and beggars; 30,000 drunkards found so in the streets, who could give no account of themselves; 180,000 habitual gin-drinkers; and 150,000 persons subsisting by profligacy.

There have been at all times, and will be, hordes of predatory parasites who infest and infect the community. They hover on the outskirts and prey on the fears and helplessness of the more exposed and feebler members; or they penetrate into the vitals, and twine like the tænia in the very track and trail of nutrition and strength; or they insinuate themselves, like the cysticercus, into the very brain. They live upon the life of others. They are the forerunners, workers, children of decay and disorganization; and form, not merely ingredients, but ferments of that putrescent but productive mass which is ever passing into new forms, ever dying but ever developing. While their name is ragamuffin and Bashi-Bazouk, and while their position is that of antagonism to law and order, and to the rights of property, and while their history is traced in blood, and plunder, and revolution, they may, nevertheless, be conservators—moral coral insects that build up our reef against graver evils amid the maelstrom of dark and unfathomable wrong and injustice in which we are engulfed—serving to educe and knit together much of what is best in human nature. Freedom has been bestowed by regicides; the wealth of patriots may be the spoil of vice, at the same time that it is the reward of virtue; and constitutional

* *Des Classes Dangereusse de la Population dans les Grandes Villes.* Par H. A. Fregier.

privileges have been the fruit of the "sacred right of insurrection," for which the conspirators were hanged. We cannot unriddle these dark paradoxes; yet if we look narrowly into the dirt and hair which is mingled with the cement by which society is kept together, we may get some clue to the mystery. We find a stinking sediment produced and cast down by the evil deeds of society; but we also find that by the Inscrutable Wisdom with which society as well as nature, all unwitting, is instinct, out of the nether-deeps of evil good may arise, if we will read the lesson aright. Ours the responsibility ; and yet aping a magnificent goodness, we are too apt to look upon these tabooed classes as canker worms; they are dreaded, proscribed, extruded, and constitute, alas! those outcasts to which the charities of the philanthropist and the glad-tidings of the missionary scarcely reach. Society bars her heart and her door against them, whether they be present or potential blackguards. They are poor, and idle, and homeless; they are ragpickers, vagabonds, impostors; and in return for this neglect, they eat, like the teredo, into the very framework of society, and seemingly threaten the whole fabric with ruin. They are Arabs and nomades in a settled land; they corrupt and taint by their presence what they do not ravish nor destroy. By day they are met with in dark, dismal, and dirty purlieus; by night they haunt the broad and open streets of our cities, and elbow their more fortunate fellow-mortals. Then we meet them straggling everywhere. Once, loitering homewards, seduced into late hours by a friend's overflowing hospitality in the West, we arrived at Waterloo-bridge about two in the morning. A few paces off the Strand an old umbrella tent was pitched, and beneath its imperfect shelter (for the wind blew wintrily and wildly through a chink in the roof) an ambulatory canteen had been established, presided over by a withered old woman. She sat with her back to the storm, had her frowsy bonnet secured by a kerchief under the chin, and only paused for a second in her duties to welcome us, as we announced our intention, urged by curiosity, to breakfast with her. We were luxurious, and had a cup of chicory-coffee and a muffin, ("La, sir, yesterday's muffins, they be you gentry's off-fals,") and a long talk, and paid our twopence. While we listened to our hostess's opinions upon roast chestnuts and her domestic sorrows, as to the rent in the tent, a little panorama—little in detail, great in significance—passed before our eyes :—First, there came up to the stall and partook of the hot cheer two unshaven, tattered men, with a bludgeon and a bull-dog, and it needed not the steady gaze of a passing policeman, or the significant glance which passed between the two men as the officer of order came near, to recognise the special category to which they belonged; next, a half-naked gamin sidled up to the verge of the tent, and, we suspect, abstracted a "tater" in its jacket; then flaunted past, and towards the bridge, a parti-coloured "sad mystery" of a fallen woman, all crumpled and crushed and sodden; and, finally, reeled along a trio of revellers, shouting ten to one upon Cribb or Eclipse, or some such thing. This while we sipped our coffee.

But these, after all, are not the truly dangerous classes. We do not fear them, nor write their history. It is true that they are burglars, and prostitutes, and pilferers, and gamblers, who outrage decency and honesty; but their orbit is narrow; the wounds they inflict upon society are speedily healed, and they themselves are quickly disposed of in a penitentiary, a prison, or a dead-house. The objects of our present inquiry move over a wider area; in fact, no community, no home, no heart, is safe from their influence. They may creep into the soul upon the breath of rumour, on a time-honoured prejudice, with our blood heritage. They are the timid, the irresolute, the superstitious, the eccentric, the wayward, who possess sufficient acuteness or plausibility to protect them from the imputation or consequences of folly; but are incapable of discharging adequately the duties confided to them: who mar or leave unfinished all they undertake; who interrupt, or clog, or render nugatory the world's business, the plans of the wise, the promptings of the philanthropic; who, without malice or machination, even without a clear conception of their fatal defects, spread defeat and misfortune and misunderstanding wherever they go; and, like the physician who carries infection to those who seek his aid, they are most dangerous where they are most trusted, and where they are most solicitous to be worthy of the trust. They are the precocious who exhaust; the perverse who misuse; the romantic who idealize their powers; the incompatibles who are neither in harmony with themselves, their families, society, nor the world; the standstill, tardigrades they have been named, past whom rushes the stream of improvement and amelioration without notice or advantage; the fast who endeavour to outstrip time and enjoyment and experience. They are those who carry with them, or acquire, those taints and corruptions which affect the exercise of the natural powers, and react upon the minds of others. Were these materials positively morbid, or palpably noxious, they would be eliminated from the class of which they are a grievance and a curse. But they may preserve many of the features of sanity and health; they may be distinguished by accomplishments or dexterity; and they must remain like moral fomites—the sources of ills which they do not feel nor fear; the leaven which vitiates every particle of the community, which in a great measure renders it the heterogeneous, heaving, jarring mass which it is—the stuff which madness and mischief are made of. That evils, momentous evils, must arise to the tone and working of every sodality, and which are perhaps incapable of remedy, from the presence and influence of such members, may be exhibited in various ways. The *Comptes Rendus* record that in six years, of 76,613 persons condemned in France, 11,387 lived in idleness and indolence, and seemed to have been seduced into crime rather by intolerance of exertion than by baser motives. The mere hard drudgery of life, and the power to engage and persevere in it, are proof-armour preservatives against ourselves. Brierre de Boismont devotes a chapter to suicide consummated from ennui, tædium vitæ, tiredness, not only of the cares and sorrows and privations, but of the very act of existence; and reveals that even such busy but perhaps overburdened minds as those of Napoleon and Dupuytren did not escape this disgust of life. Of the timid we learn that in about 6600 lunatics, 124 had yielded before the shock of terror or the corrosion of habitual fear. The irascible and the incompatibles in one year displayed feud with their fellow-men in four poisonings, sixty-one fire-raisings, one hundred and four assassinations, forty-one murders, and twenty-eight involuntary homicides. Of the vain, the extravagant, it has been written—“ La religion s'attache sans cesse à combattre ces deux mortels ennemis de l'homme.” These illustrations apply only where such tendencies crop out in so formidable a fashion as to necessitate interference, and leave unnoticed the minor but more perilous manifestations which simmer and seethe and sap, and are chiefly recognised by discord and disturbance and moral unhealth.

Society should ostracise the pusillanimous. They are the mainspring of panics, the generalissimi of retreats, and the pigmentum nigrum of all heavenly pictures. Timidity is a moral delirium tremens. The pale-visaged, hair-blanching, white-blooded, shivering terror is a scarecrow, and may provoke laughter as well as warn of danger. But our thoughts and character, and the current opinions and habits of mankind, are interpenetrated with the feeling of apprehension in a thousand different ways. It is the bond between the slave and his master. In the imbecile individual, and in the inferior subjugated tribe, the vague exaggeration of power, and the submission and subservience to the omnipotence and destiny thus conjured up, completes and fetters the chain. There are in this free country hundreds of hewers of wood and drawers of water whose services are secured upon the same bases. There is a worse slavery among the free and the independent. Man fears his fellow-man, and yet families are cemented together by fear. Man fears himself and his own weakness, and leans upon inferior natures for support, or seeks, in resorts and resources which he loathes, to escape the dark shadow from his own deeds or desires which falls across his path. Montaigne says, the thing in the world I am most afraid of is fear. Men cultivate, play with the horrible when it is not real, and enjoy the alarm and anxiety of a Sensation drama with the constitution and conscience of cowards. The sanguinary school of literature is founded and flourishes upon craven susceptibilities. Our criminal code is designated the terror of the law; the genesis of thought is effected under the salutary discipline of the birch; and despair and despondency are elements in many religious systems. Nay, with the harmony and hymns which murmur about our cradle bed there is mingled the growl of a bug-a-boo, which is to serve as an anodyne or a primary lesson in ethics, and to make the baby good.

It is an old belief that you come to resemble that which you love, and that a feeling of beauty shines in light and loveliness through the most gnarled face and the coarsest hide. But fear turns you into what you dread. The horrors of vampyrism multiplied victims; and the dread of spells or incantations, of the evil eye, or of the second sight, or the desire to escape from that dread, made witchcraft a reality and a pestilence. Reid said, epigrammatically, that a suicide rushes into the arms of death in order to avoid the horrors of his countenance; and truly the fear of death is fatal to life. Were the people numbered on the eve of an epidemic, the safety of the bold and the indifferent alone could be predicted. Mortality is increased during pestilence by the contagion of alarm, which accompanies step by step the contagion of disease. The panic-stricken die first; then the feebleness and exhaustion of despair predispose to infection; and the multitude fleeing from the poison breathed from the air or their fellow-men perish, like an army retreating before a conqueror—more from the accidents of moral disorganization than from the assaults of the foe. But under such a crisis many die from simple terror unscathed and untouched by disease. During cholera we knew of several cases in which a vague foreboding or a conviction that their doom was sealed, ended in death; and where the victims of an emotion were registered and consigned to the corpse-pit as destroyed by the prevailing sickness. Were we in the mood for proving propositions, we might quote the immunity enjoyed by those who wear the armour of fortitude and faith, and the history of the ninety Sisters of Charity who ministered for months at the death-beds in the darkest and dirtiest shades of Paris during the decimation of 1832, and did not lose one of their band.

Wellington won a victory by seeing a movement of Soult's arm through his glass, and interpreting it correctly.. But battles depend upon even more impalpable trivialities still. The white feather is not a fiction. It is difficult to conceive an army of poltroons ; but it is easy to picture the mighty mass, woven and welded together by discipline, and habit, and imitation, paralysed by one craven heart, and then rushing to defeat because their commander did so. Many conquerors have boasted that constitutionally they were cowards. A little less vanity and a little more irresolution and Marengo might have been converted into a rout by the cavalry following the white plumes of the King of Naples, and the sun of Austerlitz might have set in shame as well as in blood. Murat had momentary panics ; he trembled till the carnage began. It was, perhaps, in the madness of terror that he displayed his chivalry and foppish bravery. Le beau Sabreur, however, stood up stiff and straight, and bared his breast to be shot. The notion that every man who wears a cartouche is a hero is a pleasant fiction which protects many a country. A census of the potential runaways and renegades in a crack regiment would be a valuable contribution to immoral statistics; but until we secure such data, we may glance at the dissolution and disappearance of great armies after a defeat, and at the delirious retreat in the Russian campaign when the fugitives turned at bay, fought in horror and desperation in order to gain time to hurry homeward ; and at the fatal facility with which even men under fire are thrown into consternation and hear the echo "sauve qui peut" in every dubious sound. They say, the white-livered say, that there is a skeleton in every house ; we know that there is a spectre in every ruin and dark lane, and we suspect that there is a haunted chamber in every heart. Conceive the whole heterogeneous mass of mankind ague-struck morally ; different in all things, disagreeing, disputing, quarrelling about everything, but resembling each other in cowardice and apprehension. They would be diversified by the degree of trepidation ; but the only sneer would be levelled at the man who was exempt from the common scourge, who did not share in the universal affliction. But yet the feeling need not explode into frenzy, as during the plague in London, when the terrorists rushed through the streets, shouting "Woe, woe," else it might spread like darkness over creation ; it might penetrate every dwelling, be mixed up with the work and the worship and the meetings in the market-place, if such there could be in that doomsday ; and it might creep into the soul, and leave its trail over every hope and affection ; and might make the best and kindest selfish and sullen and solitary ; and might flicker in every eye and quiver in every limb ; till all men dreaded each other, and thought of nothing but death and punishment, and society would be shaken to its core. And there is a chapter in the world's history in which these phantom words are dark, cold, appalling facts. The world was in this plight in 1000, when the chivalry, that in a brief time, after better feeding, and having a motive, rushed to Jerusalem, and the churls who stayed at home were in a catalepsis. It was no sudden commotion or consternation. There were preparatory centuries of misfortune and civil discord and ignorance and superstition. There had been what is even more prolific of fear, want and starvation to an extent suggesting the shedding of human blood, cannibalism and every distempered appetite, and human flesh was sold in the markets. Prophecies of doom and destruction and of the impending end of the world circulated from country to country, from mind to mind, dread and despair were universal, and all men prepared, according to their character and conscience, to die. Vengeance put up the sword. Then was proclaimed the truce of God. The serfs were set free. The fields were left without culture. All that was rich and rare was placed upon the altar as a propitiation, or as worthless to doomed and dying men. The population lived in the churches, where the song of praise or the Miserere never ceased; and even while the globe was supposed to be reeling towards nothingness, the basilicas were built or repaired. Four kings and thousands of nobles retired to the cloister as men whose work was done, and who desired to perish under the shelter of God's house. There was an anticipation that all labour and industry, all order and government would be abandoned, and that the apathy and anarchy of panic would realize the foreshadowed extinction of mankind; and a crowned emperor was sent back by a cowled monk, from the cloister which he coveted to the throne and responsibilities from which he had fled, "to watch with all his soul, in fear and trembling, over the safety of his empire."

Now, what is this malaria which rises from swamps and charnel houses and man's degeneration, and which, although it never blazes out bravely in a will-o'-wisp to terrify beldames, is mingled with every breath we draw, creeps in wherever there is a weakness or a wickedness, chills and unmans us, and poisons life and vigour and hardihood, and sicklies resolution o'er with "the pale cast of thought"?

It is a conservative instinct, and makes hares travel with their ears turned backward, and men prudent and cautious. It is a sliding scale of prudence, pusillanimity, panphobia. We cannot escape from it in sleep. There is intuitive fear, and so omnipotent, that we have known a man terrified out of his senses in and by a dream, and remain a lunatic for life. And there is in such dreams a horror and mastery which colour the waking imagination. There are unfortunates who never sleep but to start or to be gripped and garotted in the loathsome embraces of an incubus, or who wander about staring and gibbering and scaling roofs, the phantoms of the sleep-world. All these sensations may be traced to the outer world. The panic may be the calming down of veins that run with passion, as well as wine; and the ever-recurring sensation of falling from a height, or of drowning, is but an expression of heart disturbance. A friend of ours proved statistically that since the increase in the number of affections of that organ, panphobics had multiplied. Perhaps we hear less of nightmare and the apparitions for which it was mistaken, because the night of hot suppers has passed away. It is recorded that night terrors have become contagious, and there was certainly an epidemic of somnambulism in 1642. There is epidemic fear in every commercial crisis, in every rush upon the Bank, in conflagration and pestilence, which thrills to the extremities of the body-politic, and staggers those who have nothing to lose, nor preserve, nor protect, and who, apart from this soul-quake, are indifferent to suffering or extinction. This enchanter that perils the solvency of a firm, the bankruptcy of a people, or who strikes a poor birdcatcher with a moral leprosy as white as snow, has sometimes waved his wand over a whole district.* In 1600 there were multitudes of men who in the mountains of the Jura, under a sense of terror and superstition, conceived they were metamorphosed into wolves. We have seen only one woman-wolf in our own day, and she leapt and snarled and snapt and howled as if in mad career along a Russian steppe. These lycanthropes and cynanthropes went on all fours until their palms became paws; they emulated the destructive appetites of the brute whose name they bore, and murdered children; they crawled about in the night, hunted in packs, it was confessed, and conceived that they held a satanic saturnalia among the rocks. They were perfectly sincere, they courted the sanguinary persecution to which they were exposed; admitted every accusation, repudiated all excuses, explanations, alibis; solved the difficulty as to their non-resemblance to their kindred wolf by declaring that the hair of their skin was turned

---

* The wand is like harlequin's, and transmutes everything it touches. Pope's picture is not a caricature of the moral pantomime :—

> "Remembered throngs on every side are seen
> Of bodies changed to various forms by spleen.
> Here living teapots stand, one arm held out,
> One bent; the handle this, and that the spout:
> A pipkin there, like Homer's tripod, walks;
> Here sighs a jar; and there a goose-pie talks;
> Men turned to monkeys; as great Fancy works;
> And more turned bottles cry aloud for corks."

inwards. They were imprisoned, tortured, burned, and perished to the number of 600. We recoil from such a holocaust, but what were the terrified human species to do ? The presence of one harmless lunatic in a district spreads consternation far and wide ; and if it be possible to conjure up the idea of hundreds instigated by the wildest phantasies, the most bloody propensities, rushing through every village and homestead, we can understand that self-preservation or a terror as powerful as that which called up the delusion, would lead to extermination. Then their story was believed, and they were hanged as genuine wild beasts. The judge, the court, the accused and his accomplices, the strong man and the weak, the parents whose children had been destroyed or were daily exposed to danger, the lonely shepherds in the deep valleys, the town-bred population who gathered together to see the weird wolf die, were all paralysed and panic-stricken, and hushed and traitorous fear brooded over the land. Such ravening creatures still crawl the earth, for no form of human error ever dies out. Cows, cats, a lion of the tribe of Judah—we have had charge of a whole menagerie : and although in accordance with the law formulized by Esquirol, that the timid and suspicious will fear that which, under existing circumstances and in relation to temperament, is fearful and formidable, we would not be transmuted into dishonoured bills, a crushed locomotive, or a drugged racer, there exists no guarantee against a panphobic metamorphosis.

There is a great deal of muscular wisdom as well as muscular Christianity abroad in the world, but there are thousands of men who would face the Redan, or a court of law, or the bitterest calamities, who would blench before a never-ceasing secret fear, a shadow that was ever present, that passed between the heart and its joys, its very thoughts. There is endemic fear—the Timoria which broods among the stagnant marshes and effluvia in Sardinia, and makes its onset like an intermittent ; but the cold stage is one of alarm and debility and tremor. There is hereditary fear. We have traced it through three generations : and if you gaze attentively at the statue before you, cold and white as the marble ; quivering like the aspen ; the almost imperceptible pulse ; the tumultuous beating of the heart ; the panting, the giddiness, the indistinct vision, the syncope, you can comprehend how men die of moral shock, and how powerful and permanent must be the effects upon the system. Theory tells us that the intense moral perturbation reduces the capillary circulation of the brain, suspends the elimination of nerve-force and mentalization, abrogates volition, enfeebles muscular motion, renders our acts instinctive, and lays us open to fascination, catalepsy, and organic changes. Popular observation has shown that the hair may be blanched in a single night, under the fear of death. We have recently examined a girl, whose eyelashes, whitened in a few hours during violent excitement, contrasted strongly with her chestnut hair. Walshe is an authority for believing that fear and other mental affections induce cancer. Sir A. Cooper attributes three-fourths of the cases of scirrhus to the same cause; and we have observed that scirrhus of the rectum, pylorus, and uterus is associated with melancholia and panphobia. Corvisart first announced that fear was at once a cause and a consequence of structural cardiac affections.

*The Tainted.*—There is certainly something in being the descendant of a hundred kings; even if these were the unfortunate Stuarts who never slept in a bloodless shroud. You may be a bit of Cæsar; the quintessence of the grandeur of purpose and deed which has shone out on the world for a thousand years; you may be the very life which pulsated and ebbed when Boadicea's chariot went down, or when the cry was "Jerusalem, Jerusalem!" There are traditions that such peers and paladins bear crosses and fetters and bloody finger marks on their body generation after generation. There are strong grounds for believing in the transmission of curses. There are races who seem the scourges of human kind: Borgias, misanthropes, who work out a destiny fraught with sorrow and suffering to all who come within their baleful influence. But it is a matter of professional experience that the stalwart arm that struck for England and St. George at Agincourt may have trembled in palsy and sent down the malady, as well as the sword, as heir-looms to the present day. The difficulty has arisen not to demonstrate that we inherit qualities and peculiarities, but what we do not inherit. You can trace a wart on the nose through three generations, and night blindness through six. The Apollo-like form, the judgment, the very delicacy of touch or taste, the infirmity or absurdity, of which in turn we may be proud or ashamed, may be the mere repetitions of characteristics which distinguished or degraded our race ages ago; and the very thought or opinion to which we tenaciously adhere as our own, and for which we would die as others have died, may be nothing more than a cast-off habit originating in a discreditable ancestor. There is an individuality within us which neither training, nor circumstances, nor will of ours can impart or entirely extinguish; but the very strength and indestructibility of this impress suggest the suspicion that it is the representative, the accumulation of unmeasurable antecedents. It is matter for congratulation that intelligence and imagination or musical talent run in families; and however little complimentary it may be to a genius to be assured that he is the scion of a good stock, further speculation might stop were such capacities the only ones transmitted; but the pernicious and poisonous parts of our nature appear to be the most easily reproducible. The Austrian lip, the Norman gibbosity, or even the bandy legs and the crooked back which mark certain families, might step into the world and out of it without much comment; but when it is established that every attitude which we acquire, every feeling which we cherish, every habit which we nurture in order to rule and ruin us, every mode of thinking, may connect us not merely with the past, but will inevitably reappear in our descendants, and must, through them, affect for good or evil the community in which they move in ever-widening circles, the subject assumes a new importance. It is curious but true that the furious passion, the pettifogging spirit, the very screwed-up, stern mouth, the contemptuous nose now extant, may touch the confines of humanity and die out in the last man. Hill has proved that crime is hereditary; that theft is a chronic malady passing from father to child, and breaking forth, like the passion for savagedom in the parentless and reclaimed Indian children; where the progenitors were hanged while they were in the workhouse nursing; and where they never took degrees in Dartmoor. The population of our workhouses is permanent, and certain hospitals are crowded, by what may be called a valetudinarian caste. It is not necessary to enter upon the impracticable question as to how far transmitted tendencies to evil should affect responsibility, or whether we should not treat ticket-of-leave men as we do inferior races of cattle; but we cannot blink the consideration of the general effect which must be produced by laws of organization such as Baillarger has described. It is not simply whether the taint runs uninterruptedly or intercurrently, whether it involves both sexes in the same proportions; it is not the broad harsh outlines which strike us most. But under such premises it is quite possible that we are not dealing with a criminal of the time at all; that we are struggling with a prejudice, an antipathy as old as the Heptarchy; that our fate or his fate may hang upon a subtlety or refinement, or a falsity, which grew up in a pagan mind; was diluted by Mohammedanism, and is now sublimated by some Christian theory of which we never heard. And then the indomitable spirit of our ancestors may warp and enchain our own will; we may look through it and get no further in our progress than the Middle Ages; we may revolt against the thraldom and think with the world as it now is, but feel with the world as it was in the days before Queen Bess: and we may live in a struggle, and a dubiety, and a nullifidianism; between a proclivity as obscure to us as the beating of the heart, and convictions which we have realized and are as clear as conscience. Again, how can we know,—and the uncertainty is a source of constraint,—that the laws which we must obey, the institutions which are the centres of mutual compact and co-operation, and the customs which interweave and embrace the brotherhood of man, are not less the expression of free and pure reason than of taints and tendencies which are akin to disease? We have a guarantee that wisdom and anxiety for the general good presided over the gradual development of these safeguards of society; but it is highly probable that the pertinacity with which modifications in these are opposed, the *nolumus leges mutari*, is due, not so much to respect for antiquity and our forefathers, as to a transmitted tendency to think as they thought. Penalties, enormities, and absurdities blot our code as to offences which have ceased to exist, or ceased to be believed in: but these are vestiges of former general beliefs which are now local; of opinions formed by wise, but lingering with ignorant men; and which have left the feeling of dread where the trust in witchcraft has passed away. Johnson believed in second-sight, and we have met men who do so still, and who have their dark hour. They so believe in virtue of a hereditary law of their nature which they cannot defy nor uproot. It would not be profitless to analyse a human mind and to apportion what is due to acquisition and what to innateness; and although the estimate would be but an approximation, it would be found in each case that every man brings to the commonweal much over which he has little control; much that leads in directions which are determined by derived and tyrannous instincts; much that is formed or deformed and coloured by his constitution and temperament; much which is a penumbra, a symptom, a premonition of coming disease, or the relics of diseases which may have decimated and desolated his progenitors. If a man make a will while the aureole of phthisis, or the eclipse of heart disease, which are both hereditary affections, occupy his mind, can the terms be expected to be the same as if the sober understanding guided the pen? Nay, more, the self-deceptions which the hidden processes of decay, the dynamic influence of pain or irritation, the poisonings of wine, or food, or bile, or urea may generate, influenced as all these are by transmitted conditions, must approach, if they do not betray, our best purposes, our most profound reasonings, our greatest discoveries. Poetry has been inspired by Rhine wine and gin-and-water; philosophy, and criticism by opinion; and pulpit oratory by pain: and all in men who were victims to hereditary taint, who hovered on the brink of mental disease or plunged into its abyss, and who have influenced their generation more than most of their contemporaries; and how much through the strength of transmitted disease, who shall decide? But that these characteristics multiplied, that it was the anormal and spasmodic ingredient which was thus propagated, the romantic robbers who imitated Schiller's fiction, the sentimental misanthropes who affected Byron's least manly vice, and the second-rate ranters who presented Hall's peculiarities without a spark of his impassioned oratory, sufficiently prove. It is asserted that the face of the dead sculpturesquely brings out lineaments of some far-off prototype; so in the moral death of mental alienation, when the natural and noble qualities which gave a name and a place, and which cause a man to stand out in relief from his fellows, are blurred and blotted out, he is fused into an amalgam of all the basest natures, or gives traces of the mould in which he was cast. But is there a peril or a penalty to be dreaded from this law of descent? The danger corresponds to the nature of the endowment. It may be a grace of person, or an obliquity of mind; an ambition which ennobles a serf, or which destroys a dynasty. The head master of an English school, the bearer of a historic name, lived for thirty years in an exuberant enthusiasm for classical literature, in intense devotion to his studies, and died phrenitic and delirious. His five sons went forth into the world, one, a poet composing in darkness, opium, and tobacco-smoke, yet exquisite in his deliverances; another, a drunkard, an epileptic, and a painter; a third, a clergyman who lived in utter solitude; a fourth, a dissolute man about town, of feeble frame; who was a voluptuary without pleasure, wicked with a great reverence for what was good and beautiful, and who lay in bed for weeks in sheer cowardice of the discomforts of activity; the fifth was a burly braggart, who sought distinction in the bar of an inn, and found sufficient excitement in ale and skittles. The first danger from such propagandists was, that their homes would become the stage of their own Nemesis; the second, that they should increase and multiply and form alliances and ties in the webwork of an old country where lineage is interwoven with the thinkers and rulers and representative men of the day; the third, that with their undoubted gifts and genius, there would flow along the channels which they might command in the flush of youth, or the waning force of age, wild fancies and forms and gloomy fanaticism and bacchanalian orgies, which might have the flavour of genius without its vigour or its purity; and the fourth, that they would dwindle and degenerate into drivel and delirium. The doom of this race is cast; two have been in asylums, the others are either childless or their offspring are idiots. This is a dark picture, the darkest that nature ever furnished, but solely because the inherited misfortunes, the errors, the failings of a dozen lines have happened to converge into one. If these pollutions, these sources of disturbance and degradation, be ramified in a less intense degree, but throughout a community, a conception may be formed of the composite influences which bear upon the human mind from the past, impair its independence, and place in jeopardy the issues of the most insignificant as well as the grandest efforts.

We have read of mad kings and mad philosophers, but they are innocuous compared with that politician who acts as a fate; who is heir to a vicious organization; and is merely the last of a series of changes which may reconstruct or revolutionize the people he governs. It is marvellous how far such pulses may reach and be appreciable: the original element remains amid all the admixture of different and conflicting natures with which it may be brought into contact and interfused. And if it be remembered that the deepest and most permanent impression is made by the mothers, and that the offspring are copies of the feebler nature, though perhaps the purest, we come into contact with another source of deterioration. It has been demonstrated that the insanity of the mother is more frequently hereditary than that of the father, in the proportion of one-third. Of 453 lunatics, the taint descended in 271 from the mother, in 182 from the father. Secondly, it has been proved that the taint affects more children when issuing maternally than paternally, and in the proportion of one-fourth. Thirdly, it has been proved that derangement in the female is more frequently transmitted to the female than to the male children.

In the hope of bringing clearly before our imagination the possible extent and complications which might arise from intermarriage, we have contrasted a table furnished by Quatrefages, of the mingling of the three races, white, black, and red; and the names appended of the different shades and varieties of colour and constitution; with another constructed from our own experience of the union of diseased, nervous and healthy members of our own branch of the human family. The formula is not exhaustive, but may serve as an index of what may be seen in a third of a century, and of how far a people suffers from an individual.

1. A madman marries a sane woman; a son and daughter are insane, one stammers, one is phthisical; several are healthy.

2. A sane man marries a mad woman, a pyromaniac; a daughter and granddaughter and a son are insane, and confined with the mother.

3. A maniac, with lucid intervals, marries a melancholic, whose hard fate induces dipsomania. They are childless.

4. An eccentric, passionate man marries the daughter of an eccentric pietist. The issue was a daughter, who was allowed to die of abstinence, while labouring under delusions connected with uterine disease. The son married the heiress of an odd, sceptical millionaire. One child was born before the father died insane. That child has married a robust, well-constituted man. She has had two attacks of puerperal mania. How far her children have inborn strength to resist the family predisposition and the battle of life cannot be predicated.

5. An idiot was the representative of parents apparently healthy, one of whom was the child of an eccentric, who was the son of a madman.

The original characteristic may be detected in—

The product of a Spaniard and an Indian or Mestisa.
" " Mestisa and a Spaniard or Castisa.
" " Castisa and female Spaniard or Espagnola.
" " female Spaniard and a Negro or Mulatto.
" " female Mulatto and Spaniard or Morisco.
" " Morisco and female Spaniard or Albino.
" " Albino and female Spaniard or Tornatras.
" " Tornatras and female Spaniard or Tentinelaire.
" " female Indian and a negro or Lovo, &c. &c.

There are royal races, in which it is obligatory to marry relatives. Tribes that are stationary, secluded, separated by mountains and morasses, and by the more stupendous barriers of blood-feuds, did the same thing. The purely domestic, in contradistinction to the social habits of our Celtic element, their clanship, their mode of government, made every man his neighbour's cousin; and this may explain the great similarity of disposition and the great lack of originality observed among their characteristics. What local difficulties effected, in their case, the pride of descent among aristocratic families, the interests and accidents of class privileges, professional ties, pecuniary necessities, and the absence of railroads, have brought about amongst the more favoured inhabitants of the plain. If you examine the registers of a country town, or fall upon a genealogical gossip, you will discover a closeness and intricacy of consanguinity: a web of cousinship which makes the world akin. We have, in this old country, been breeding in and in ever since the Norman conquest, and transmitting consumption, and scrofula, and peculiarities, and the natural character, as if it had been desirable to do so, and to multiply sources of destruction. It is, moreover, difficult to get above or out of this type or stamp. We are trammelled by our very strength and boast; our ancestry keeps down what may be original in us, and mingles with and modifies our acquired knowledge and tastes. The real difficulty in the pursuit of knowledge is for a man to live on and trust in his own resources. The beneficial influence of revolutions is far less in changing the mode of government, than in giving mankind a new start, and in uprooting hereditary tendencies, prejudices, and habits.

*The Credulous.*—How much of a man's convictions are made up of credulity? How much of the world's opinion and wisdom and miseries are personal convictions, uninquiring faith; a wish to believe; a colouring cast over every thought and object by the hobgoblins of childhood or the superstitions of old age? It is not possible that a physician trusting to Parr's pills and such elixirs vitæ, can avoid thinking and seeing through the mist, and having his judgment warped and magnified like the giant figures of the Brocken. It is not possible that a judge who believes in spiritualism—and there are such judges—or in his newspaper, or the physiognomy of a witness, can divest himself of the blindness and the perversion of evidence consequent upon such a state of mind, and see naked truth. But the world could not bear the goddess unveiled. Our castle builders would be shorn of a part of their happiness, of their being, by such a revelation. Were they, however, to build their chateaux en Espagne on their own property, visit Eldorado in dreams, and leave fortunes only in their wills, the evil might remain personal; but, like the pores of a fungus, their plans and projects and actual structures float through many brains, permeate the densest parts of society, and grow up again in fifty places. We have travels into countries flowing with milk and honey, histories founded upon a system or a crotchet, and personal experiences which represent men as angels or devils. A friend of ours lived up to an income which she did not possess, and then solemnly bequeathed it by testament: another preached his first sermon on the fall, and discovered afterwards that he had drawn the whole from *Paradise Lost:* and a whole circle of our acquaintances derive their knowledge of Scottish history from the inspired pages of Sir W. Scott. The biography of impostors has been written; of Psalmanazar, who invented a language; of Greatrakes, who cured the surest of deadly diseases; of Cagliostro, who made the old young, and the young immortal; but there is a chapter wanting, the history of those they imposed upon—the learned critics of the one, the recovered patients of the other, and disappointed dupes of the third. There is a harmony between every different kind of imposture and empty rumour and different human minds. For every error, as well as for every truth, there seems to be a mind ready prepared—amoral gap into which it electively fits. There have not only been a dozen Kings Sebastian who himself was killed on the field of Larache; there are not only vicarious James Flemings who aspire to the scaffold; but there has been a succession of

Wandering Jews for the last 1900 years, who believe their destiny, and are believed. Make a broad, bold, emphatic statement, and part of it will be credited; a bit, or a side, or a phase of the extravagance finds out the hole where it will be received and morticed, the soil in which it will germinate. No ideal but has its worshippers ; no cause, however desperate, but has its defenders ; no lie, however gross and improbable, but has believers ; no habit, however absurd and repulsive to nature, but has imitators. Even in Christian lands every man has a fetich ; and it would be instructive could an orthodox congregation be analysed into the component elements of the true and the traditional, of the teachings of the faith and of fancy. This tendency to the marvellous has not merely provided many with creeds, but it has peopled every stock and stone, and wood and water, with spiritualities. It has superadded mysterious to the acknowledged qualities of every shrub and star. It has surrounded us with an atmosphere of gloom, so that it is difficult to carry the eye to the clear sunlight which beams beyond, or to distinguish the forms which it may envelope. To those who desire to learn the historical aspects of wonder-working in human character, we would recommend the study of Calmeil's *De la Folie, consideréе sous le point de vue Pathologique, Philosophique,* &c. We care little for the Demonolatria. The conventional and venerated raw-head-and-bloody-bones superstition may keep up a wholesome belief in the supernatural. But there are more insidious forms of marvellousness which, with "fear of change" perplex. Whenever a plague bursts forth, instead of opposing the foe, they believe in the poisoning of the wells. There is, likewise, an adulteration phantom, and a colour madness as to arsenical green, which have taken the place vacated by slow poisoning and the aqua toffana. These suspicions inflict unmitigated misery upon the gullible wherever they are—and where are they not ?—and cast a lurid hue over the honesty and humanity of our fellow-men. But they do more ; inoculated upon the nervous or morose, they evolve the hideous and intractable compound, lunacy in which food is abstained from and inanition is courted, not in order to destroy, but to preserve life, in order to avoid drugs and poison and putrilage and the crime of cannibalism. The enormous consumption of medicine by the healthy may carry with it the compensatory advantage that it sustains a faith in the efficacy of medication for the sick and the suffering. Various forms of disease arise from substituting physic for food and air and enjoyment. Many a physician unwittingly proceeds upon the principle similia similibus curantur, when he endeavours to counteract by drugs what has been caused by drugs. This credulity and legitimate practice are equally hurtful. The belief in infection has snapped asunder the affections as well as the duties which bind man to his fellow-man; and the reign of pestilence has been as conspicuous for its credulousness, its faith in portents and prodigies, and for its selfishness, as for the wretchedness which it inflicted.

---

### Art. XII.—MEDICAL CERTIFICATES IN LUNACY.

